# Supplementary material for: Use antibiotics in cell culture with caution: genome-wide identification of antibiotic-induced changes in gene expression and regulation
Source: Sci Rep. 2017 Aug 8;7:7533. doi: 10.1038/s41598-017-07757-w (PMC5548911; doi:10.1038/s41598-017-07757-w)
Supplement: Supplementary file 1 — Supplementary Figure S1 [file 41598_2017_7757_MOESM1_ESM.pdf]

Use antibiotics in cell culture with caution: genome-wide identification of antibiotic-induced changes in gene expression and regulation

Ann H. Ryu[1,2], Walter L. Eckalbar[1,2], Anat Kreimer[1,3], Nir Yosef[3,4], Nadav Ahituv[1,2*]

[1]Department of Bioengineering and Therapeutic Sciences, University of California San Francisco, San Francisco, CA, USA

[2]Institute for Human Genetics, University of California San Francisco, San Francisco, CA, USA

[3]Department of Electrical Engineering and Computer Science and the Center for Computational Biology, University of California Berkeley, Berkeley, CA, USA

[4]Ragon Institute of Massachusetts General Hospital, Massachusetts Institute of Technology, and Harvard University, 02139 Boston, Massachusetts, USA

*Correspondence: nadav.ahituv@ucsf.edu

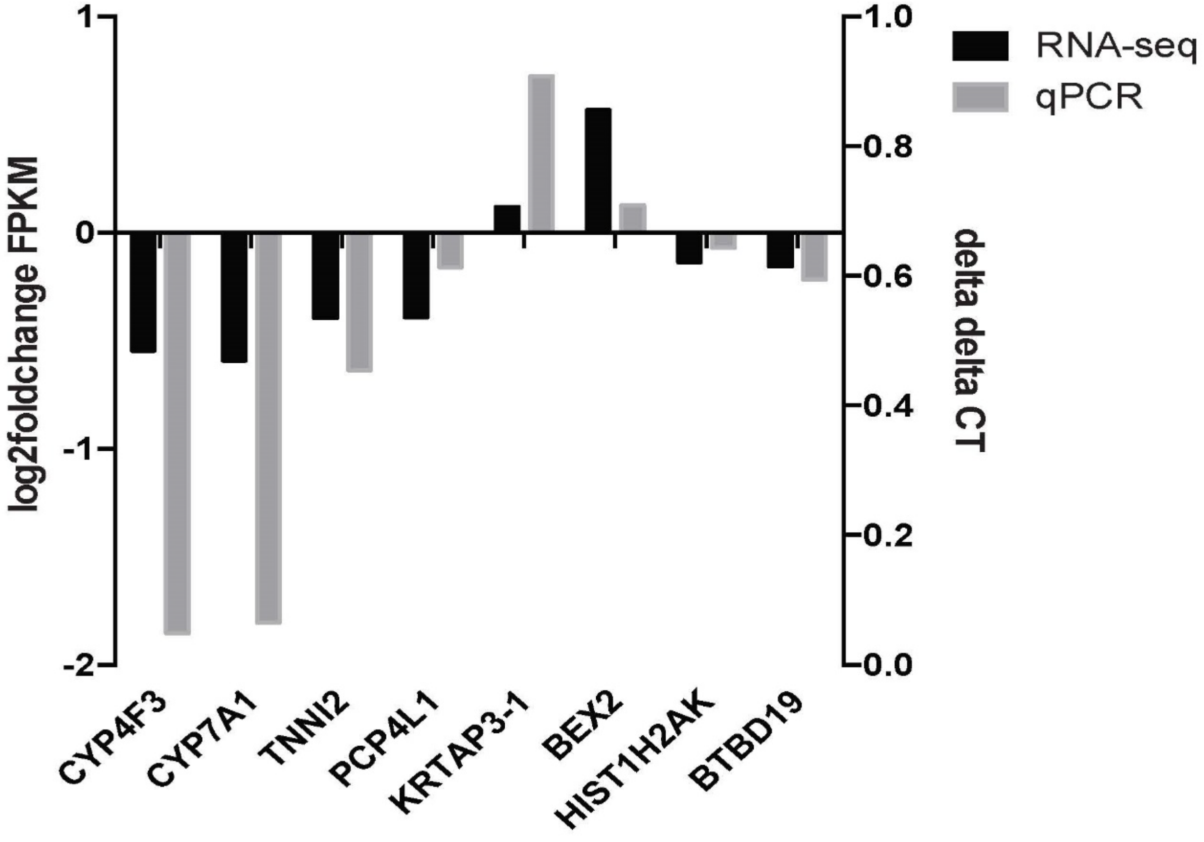

Supplementary Figure S1. qPCR validation for eight PenStrep RNA-seq differential expressed genes. $\Delta\Delta$ C_t values for *CYP4F3*, *CYP7A1*, *TNNI2*, *PCP4L1*, *KRTAP3-1*, *BEX2*, *HIST1H2AK*, *BTBD19* were normalized to housekeeping gene expression levels, then calculated between PenStrep and control conditions. These $\Delta\Delta$ C_t values were plotted in parallel with the log2fold change FPKM values from the RNA-seq results for each gene.
